# Supplementary material for: Transcriptional regulation and overexpression of GST cluster enhances pesticide resistance in the cotton bollworm, Helicoverpa armigera (Lepidoptera: Noctuidae)
Source: Commun Biol. 2023 Oct 19;6:1064. doi: 10.1038/s42003-023-05447-0 (PMC10587110; doi:10.1038/s42003-023-05447-0)
Supplement: Supplementary file 2 — Supplementary Information [file 42003_2023_5447_MOESM2_ESM.pdf]

Supplemental information

Transcriptional regulation and overexpression of GST-cluster enhances pesticide resistance in the cotton bollworm, *Helicoverpa armigera* (Lepidoptera: Noctuidae)

Minghui Jin[1†], Yan Peng[1,2†], Jie Peng[1,2†], Huihui Zhang[3], Yinxue Shan[1], Kaiyu Liu[3], Yutao Xiao[1*]

[1]Shenzhen Branch, Guangdong Laboratory of Lingnan Modern Agriculture, Key Laboratory of Gene Editing Technologies (Hainan), Ministry of Agriculture and Rural Affairs, Agricultural Genomics Institute at Shenzhen, Chinese Academy of Agricultural Sciences, Shenzhen, China.

[2]College of Plant Science and Technology, Huazhong Agricultural University, Wuhan, China.

[3]Institute of Entomology, School of Life Sciences, Central China Normal University, Wuhan, China.

[*]Corresponding author.

Yutao Xiao: xiaoyutao@caas.cn

[†]These authors contributed equally to this work

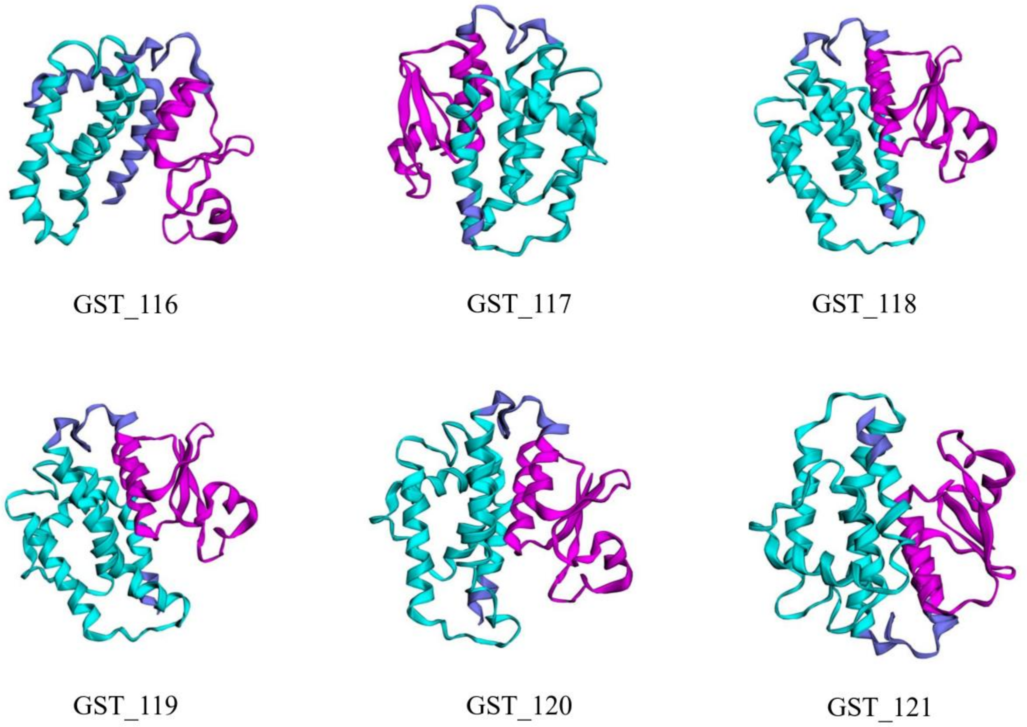

Supplementary Figure 1. Predicted 3D structure of six genes in the GST cluster. Pink, Thioredoxin_like family; Blue, GST_C_Delta_Epsilon

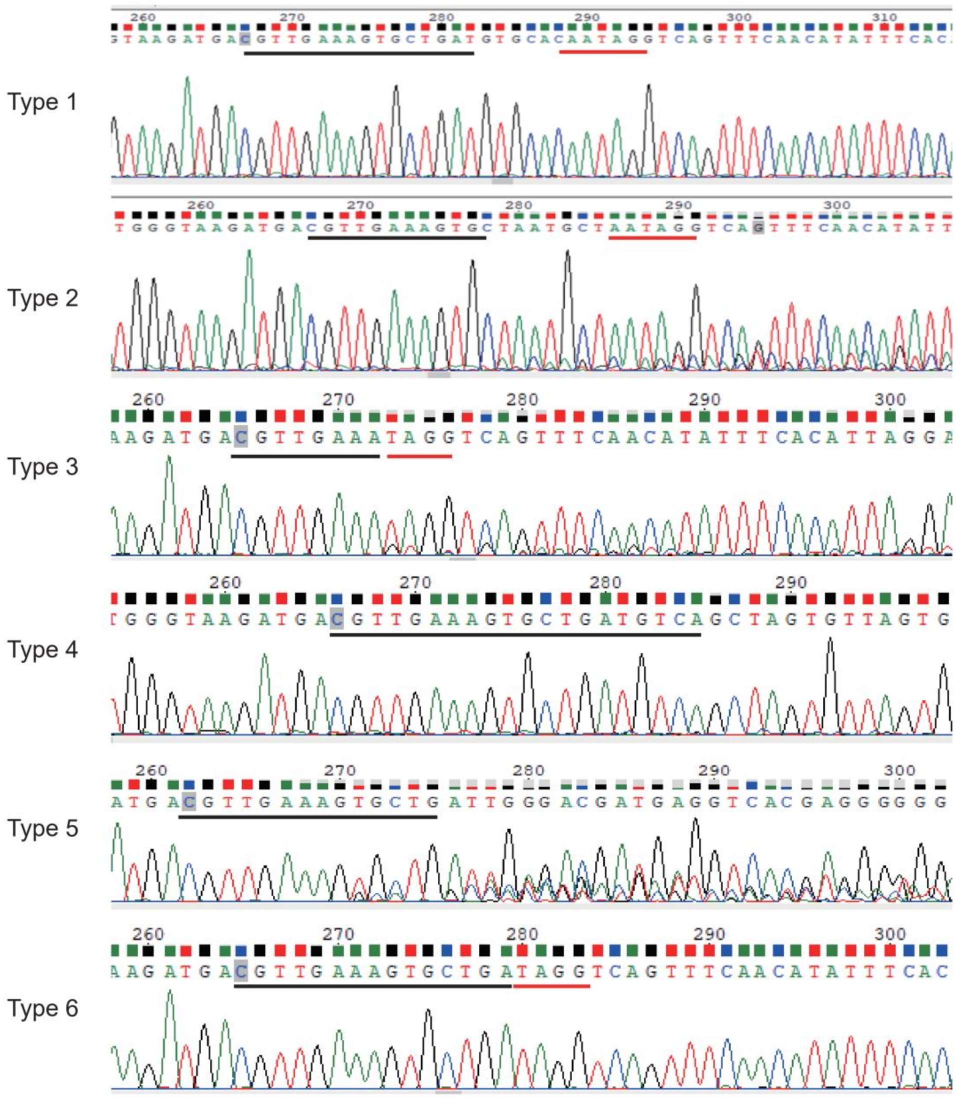

Supplementary Figure 2. Differently mutation types of GST cluster knockout. Target sequences of sgRNAs were marked with black and red lines.

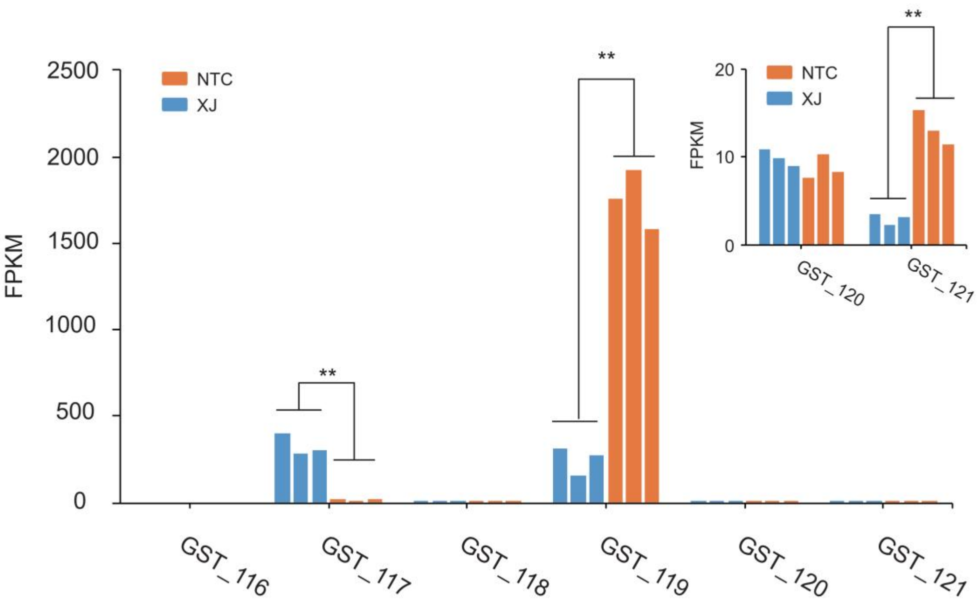

Supplementary Figure 3. Expression level (FPKM) of each GST gene in the GST cluster detected using RNA-seq data. ** *P*-value < 0.01 in Student's *t*-test.

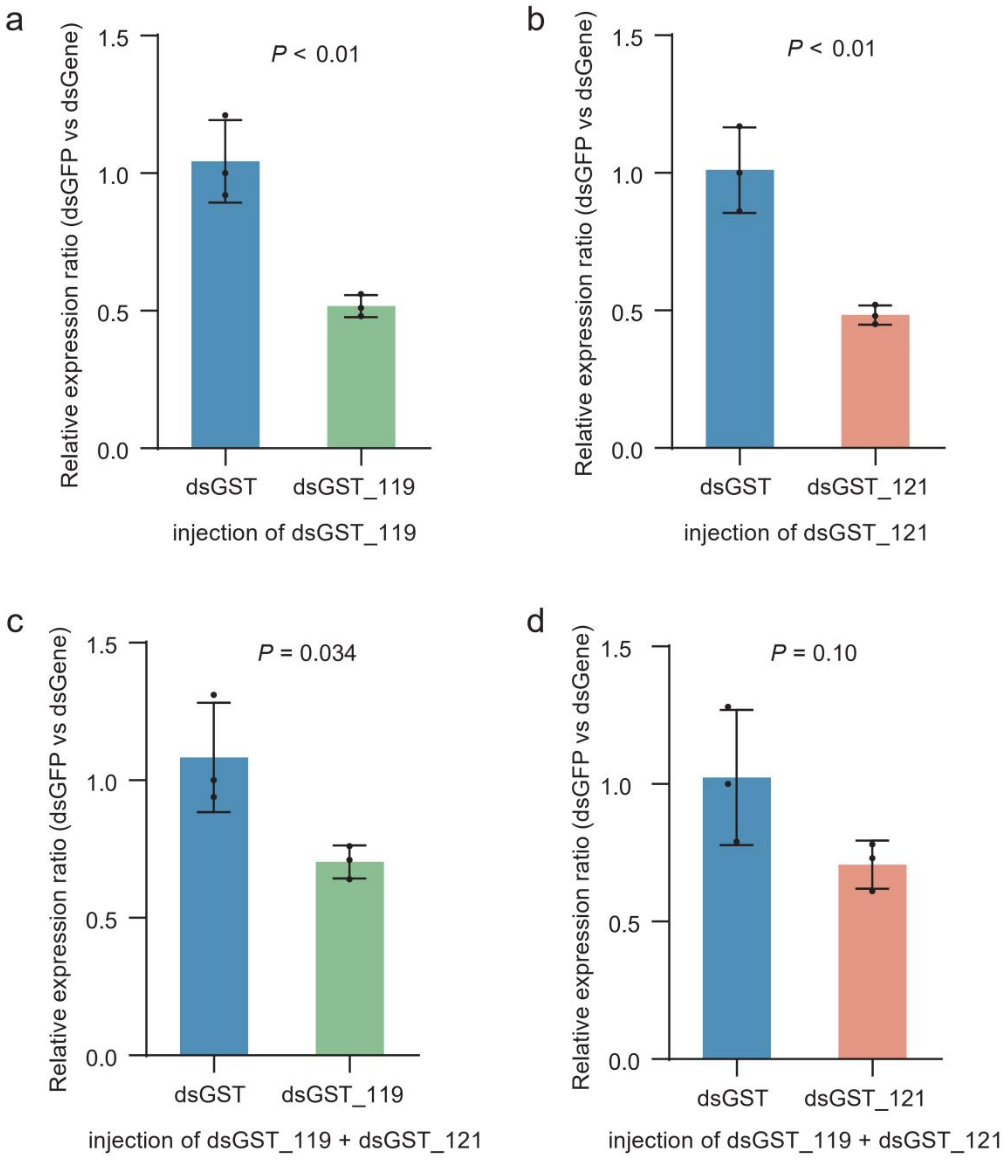

Supplementary Figure 4. Detection of the gene knockdown efficiency of RNAi. Suppression of the GST_119 gene (a) and GST_121 gene (b) in the larvae of cotton bollworm 24 h after dsRNA injection. (c and d) Suppression of GST_119 and GST_121 after co-injection of dsGST_119 and dsGST_121 (n = 3). dsGFP (n = 3) was injected as a control. Error bars represent standard deviation. Significant differences in Student's *t*-test.

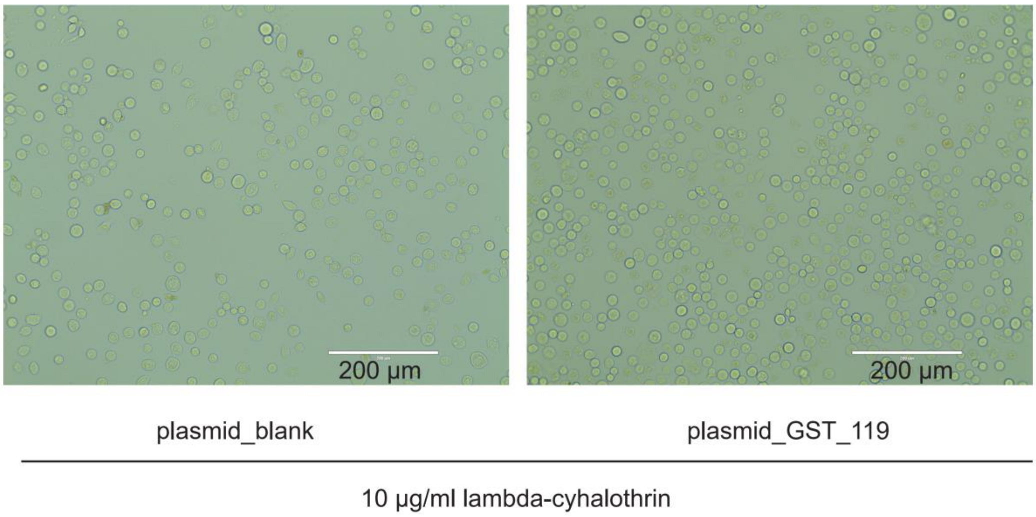

Supplementary Figure 5. Cytotoxicity of *lambda*-cyhalothrin to Sf9 cells after transfected GST_119 plasmid and blank plasmid.

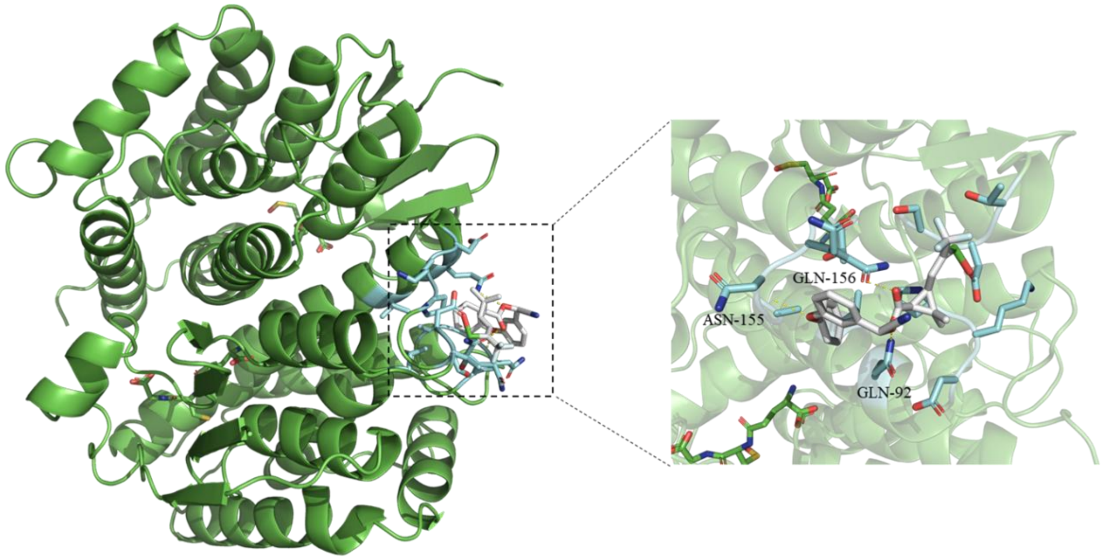

Supplementary Figure 6. Interaction analysis between GST_119 proteins and *lambda*-cyhalothrin.

```
119-KEL.txt   ....................................................................................................     0
119-SD.txt    TGTGCAAAGAGAGGAAGTAATAATAATCACCAGCCTATCCTATTATCTACATCTATGCGTACCCAGCCTATTACTAATGCGAAGAAAACTTAAAAATGGA   100
Consensus

119-KEL.txt   ....................................................................................................     0
119-SD.txt    GTAACTTCTCCAGCTTTCCCAACATTTCCCATCACTGTTCTGCTCCTATTGATCGTAGCGTGATAAAAAGTATCCTATACCCTGCCCAGGAGTATAAAAA   200
Consensus

119-KEL.txt   ....................................................................................................     0
119-SD.txt    ATATTAGTGCCAAGTTTCATTAAAATCCGTCGAGTAGTTTTTGTTTCTATAACGAACATACAGACAGAAAGACAGACAGACAGAGAGCCAGACAGACAGA   300
Consensus

119-KEL.txt   ....................T GTTC A T CA AAAA ACAAC TG A GA  AA ATT  GG T GGTT CA A A A  A AC   TA C G    80
119-SD.txt    CAGAAAGACAGACAGACAGAC  AAAT T A TC TTGC TTTTT GC T AG  CG TCA  AA C CCCC TG T G T  T GA   AT T A   400
Consensus                         aa   t t c a   a    g  a c  aat  a   ct  t a    c  a a a at tt  aaat  a tt

119-KEL.txt   TCT G TGG  CTAA ATA A TG TAACT A   T C G A A ATC G T CTC AT   GT A T TC G A T TT  T GTC ATA  CCT  A   180
119-SD.txt    ATG A GAA  ...G CCT T CA ATTTA T   A T T G T CAA A G AAA TA   AG G A CA T T G AG  A AAA TAC  TTG  C   497
Consensus       t ca  tt    a  c a g    t ata g a a a t   g a t   a  aaa  c g a  a a t t  ct c   c   tt   ct

119-KEL.txt   T   G  GTCCAATG GGTA   ATTTTTTATT ATA A    TG CTTA CT     A AAGT T  TGGA GTA GACGATAATT     A  T    280
119-SD.txt    A   .  AAATGCCC TCAT   GGGAAGAGAG GGG G    AC TCCC TC     G CTAG C  CACC AGC TTTTCCCTCA     .  A    595
Consensus      cata ta       a    cat          t   g aacga  t    a  aaaat a    t tc     a   c         aatat gg tg

119-KEL.txt   TAA AATA A CG  GTTA  AG GCCA  TTTTTG C   C G TA  CCA CCTCGT G CTG TT G  CGA G GGGGAG    .  TTC  T A   379
119-SD.txt    CCC TGGG T TT  ACCT  CA ATTT  GGGCCA T   . A CG  TAT TGAGAA A AGA AA C  TTC C TTAAGC    T  CCA  C T   694
Consensus       a    t t  ttt    ga  t    tt      t tta a a  cc   a      g g   g  g aa   t c      caag ag   tt a

119-KEL.txt   GA GGGCAA G ACC GGC  G A C   CC GG . C C  GA ATTG AA  GGCCTAAG C     AT TGCTCA CA   TCTTCATGCC TTA   478
119-SD.txt    CT TAAAGC A GGG ATG  T G A   AT TC G A T  AT GCCC GC  TAGAATCT A     CA AAGGTG TG   GGCAAGGTGG GCT   794
Consensus        a      t a   c   tt g a aatt  g  a t t aa  a    a  tt        g taaag  c     g  gtg          t   a

119-KEL.txt   ATAC GC   TA  AATAT    CGT G  T TC T AAG  A ATC A ATA A TAA     TA A    GT   TG A TC  T TCA  AAC TTA   578
119-SD.txt    GAGA AT   GT  GGAGA    TAC .  C AA A GGT  T TAT G TGG G GCT     GC G    TC   GA T GT  C GGC  CGA GGG   893
Consensus          t  ttt  ct     tttg   t ca t  t a   ta t   t t   a t   ttgat  a aatt  cta  a a  tt a   tt   a

119-KEL.txt    AT A  ACAT   T CAGTTGTTTC TTT  TAAAA  C  CG  AC  T  T TTGT GC   AAAC  CT   GCC  TA T  A CGCC  TCC T   678
119-SD.txt     TA T  GGCA   G TCAAAAGGAA AGC  CGGCC  T  .A  CG  G  A .... AA   GGGG  AG   ..T  CG G  G TTTT  GTG C   986
Consensus     a  t ta    aaa g           a    at      at ag  ga  aa aa a    a  tac    ta  tct   ca  a aa a    ca   t

119-KEL.txt    TCA GTT   CTG G G  AG..  GT G GGAAC     A    G       C      T        A T A         T    C    T           776
119-SD.txt     GTG CAG   TCT A A  TTTC  CA C TTGCG     C    A       T      .        T G G         G    A    A          1085
Consensus     t   t   tttg   g a ga    aa  g t     gagaaa tagc taataca caata aactaca a c taaaacaa aca caa ataactag

119-KEL.txt                       .. C                             T                          C            CT C                    C    874
119-SD.txt                        TA T                             C                          T            TA A                    A   1185
Consensus     taaactacttcttata  c tactaatagtaatgtccgaactca gttttttaaggaagcactaaa tgacagtact  t gattgtggagattatt aa

119-KEL.txt      T    T        T                          TGTTGCGATGACAAGCAGTAATAAGATCCGCATCATAA               T  G   G    974
119-SD.txt       C    G        A                          ....................................               A  A   A   1247
Consensus     ata catc attaatat gacaaggaaaaagttatacaaaat                                      gtacaaagatt c gta tg

119-KEL.txt                C  TG                 C         CTCCATCTTCTTCCTCCTGCCCT          A CC      TTT A A    C T    1074
119-SD.txt                 T  CA                 G         .......................          C AA      ... G G    T C    1321
Consensus     aaaacaatcatg ga  cgaattatccactaaca ctgatat                           gccctgtt a  attttt  t g tcgg g aat

119-KEL.txt        TT      T C     C          C      G      C CCA      CAC                  C T                  G                 1174
119-SD.txt         CC      C T     G          T      A      A T..      TCT                  T G                  A                 1419
Consensus     gtctt  gcttc a tctt tctgtca tcgtc tactg c   ctcact   ttcttattcatgtca c ttcaggcagtccat tccatccatttttt

119-KEL.txt     A       C                        CTACTATCAGTTGTTTATTAGATT            T                                        1274
119-SD.txt      T       T                        .......................             C                                        1495
Consensus     ct aggtgtt ttcttcctctccatccat                           gatgattag ttattggtttagagaaaatataaaaaaatataatgcc

119-KEL.txt                                         A  A A             .                       G          G   C                   1373
119-SD.txt                                          G  C C             C                       A          A   T                   1595
Consensus     gtatccataacacacggttggctcgataac gt a aatacgtccc acacggaccggcttcagcggcct cggcacttc gtc acaacttgaagcgat

119-KEL.txt             CTCTGCGAAT   C     C             G                       T      A                                 GGTGC G   1473
119-SD.txt              ..........   T     T             T                       A      C                                 A.... A   1681
Consensus     tcgatcgt            aag gagct ggattttcatt gaacaaaatcaaaaaggt atgtc aatacaacaatgtgttcagaatgctggt      t

119-KEL.txt                           T        G          ........................              A  C          C                   1548
119-SD.txt                            C        A          GGTGAAGAATGATGGATTTATACCA              .  T          T                   1780
Consensus     ccacgtttttgcagagctt gggaat ttctctga                          tcatccggaag cg tgtgcct gtttcttcgcagattgc

119-KEL.txt                      T                      AGTT   A   C        A                        .   T              G  AT    1647
119-SD.txt                       C                      ....   C   T        G                        C   A              A  .C    1875
Consensus     gtgacagctcccacag atgctgagcacattgtgcggt    ggg ttg ccatggct cggcactggatcgggcag acac tctgcggatgt tc  t

119-KEL.txt    T G T  .....  G  C      ..AGA  A AATA T TTAA CCT CTT CA  CCG AC G CC..............................   1708
119-SD.txt     C T C  CGTAG  A  G      TAGAG  T GGCG A ACGT GAC TAA TT  TTA GT T TTATAGATACATGGGTCTGACATAAATAAAATAT   1975
Consensus     a g a tc     at gt tgaca     at a    t a    a   t   t  ga   a  c a

119-KEL.txt   ........................                                                                             1708
119-SD.txt    GTTTAAACCTTCTTTCAGAATAAC                                                                             1999
Consensus
```

Supplementary Figure 7. Alignment of the upstream sequences of the GST_119 gene from susceptible and resistant strains of cotton bollworm.

```
121-KEL.txt    CAAATGCGATAGGAACAAAGTAATTAAACTAGGCTTATGCTATTGACACTTTCGTGTA  T C A  GA  A      GT   TT A  ......   GA     94
121-SD.txt     .........................................................C  A T G  ..  T      AC   CA T  ACTCAC   TT     41
Consensus                                                                aa a c ct  gt tcattt  ggc  g tt      ttt

121-KEL.txt    GA AA   CA AAAG TT   C T TT TGCTC    T    T AACATA   TGT AACG CG AG            A                 TGA    194
121-SD.txt     CG TG   TT CCGC AC   T G AC CA...    C    C CCTCCC   ..G GCAC TA TC            T                 ...    133
Consensus        g  ttc  a    a  aca t a  a     aata agtc a      acc   a    a  a  atagtataaaaa taatgtttcacatggat

121-KEL.txt    CA T  A   G    T        A           C       T      ................                     T   C.......    271
121-SD.txt     .. C  C   A    C        T           A       C      TAACAGCTACAATTGA                     A   AAAAAAAA    231
Consensus        g ct tgt attt ctaagact tactcaaactg caaacat tacata                atttaaattggttcagtagtt tgg

121-KEL.txt    ...              A                G     C  A...........  C                                       T      357
121-SD.txt     GCA              G                A     T  TCTTATAATACC  T                                       C      331
Consensus         gtgaaaaagaggca acgaaccaacagagat tttta gc            ta tagatgaaaaaatacgaactgacaaacaaaaattaccat tc

121-KEL.txt                         T                                                                                  457
121-SD.txt                          C                                                                                  431
Consensus      taatgaaaagcgtttatcctg tggtaaaatcaattattctagttgatccagatttcgtgtagaagtgtatacgtcaatcaatttctcattatcgcggct

121-KEL.txt                               A                                                                            557
121-SD.txt                                T                                                                            531
Consensus      gcctattcgagccaggcttcttgcaat tgccatcataatgcaatttccatggaacttttaacgagcaatttccttgtcgatgtctgcttgcttggatta

121-KEL.txt                                                                                                            657
121-SD.txt                                                                                                             631
Consensus      attaaatttccgtctatacgtagtatgggtcatattattaaaatattttggcgtggtttcagtgtgagcggggctaaatatttaaaattgttttactggg

121-KEL.txt                                                                                               G            757
121-SD.txt                                                                                                A            731
Consensus      tacttagctacagaaaacctgcatcgtaccaccagctattgtaccactcatacgaaaaggttcatcttatattatggcttattattttccg gcgactaa

121-KEL.txt         TAAA                                                                                    T  .       856
121-SD.txt          ....                                                                                    A  A       827
Consensus      taact    agtcgacaaatgtttgcaacaaataaaataacttgccagcgattaaatatttcggcgactattataatatgtacgctttttttt aa tat

121-KEL.txt                     TA                        G                                                TTTTAGTT    956
121-SD.txt                      ..                        T                                                ........    917
Consensus      tttttttttattcacac  tgattaatttatattcattgaaat tttaacactattttcttaatttaaattcattaaaatttattttctatt

121-KEL.txt                                          T                                                            1056
121-SD.txt                                           .                                                            1016
Consensus      cggttttctataaaaagcgtgtttttagttttttttt aaactattattaatttctacttttagtttggtttttattaaagcgtaatttttaaaa

121-KEL.txt                                  C       A             C                                              1156
121-SD.txt                                   G       G             T                                              1116
Consensus      ctattatttgttttgtagaattaaaattct tttagtc atattagagaaaa ggctaaagataatttattggaaataaaaattaaaaacgagtcctgcc

121-KEL.txt                                                                                                       1256
121-SD.txt                                                                                                        1216
Consensus      ttatcgactgtagagaaaaattatagaaaatttaattaatttcttaccttatcgactatatatgaaaattatataaattaacaaaaatcatattttgc

121-KEL.txt                                                                                                       1356
121-SD.txt                                                                                                        1316
Consensus      aaattgaaaaactttttttatttttcatttcgtgcgttgtgtaatccctatatccctttcgatacaaaagaatttctaaataatttgaaagtgtataaaaa

121-KEL.txt                                                                                                       1456
121-SD.txt                                                                                                        1416
Consensus      gctttaaaacgtaaacaagcacatacccgcctaagtttgttttatggacctaaaaatgacaatattttggaagccattattttgttgccatctattcacg

121-KEL.txt                                     G              C      A                           T               1556
121-SD.txt                                      A              T      G                           G               1516
Consensus      caaagctaaactatatttttagcgacctctatc gcgagtagcagaat atgttg tgatataactggagttcactgtgctag tttgttactcgccgct

121-KEL.txt            G                                 CT ....               G  G             T              GC     1652
121-SD.txt             C                                 AA TAGA               T  A             C              TT     1616
Consensus      agatgtct acgtaaacgttttaatcgtaattcaagacttct  a    aacattttgtggagt cg tgttcgtgtctat ctagtaaggataac  tt

121-KEL.txt                                          A     A                                                      1752
121-SD.txt                                           G     C                                                      1716
Consensus      ggttcgatcgcattttaatagatggcaggcgatacgaa tacac aattttgtccaatgcatacatacaaaatgcacactaacagataaatctgtagtta

121-KEL.txt                      A                                  .............................................     1807
121-SD.txt                       .                                  GTATATGTGCCTGGATACATACATAGGTAGGTACTTATAATATCT     1815
Consensus      aaaaattgaaaaaaaaaa caacatctttcttcttatcggttcaacttaacat                                             cg

121-KEL.txt                                                                                   CTTA        A           1907
121-SD.txt                                                                                    ....        C           1911
Consensus      aactgagaacttccttttttttagtcggcaaaaaataacccttcttgaccgtccactaataaacaacattcagtattta    catttttt ttatatct

121-KEL.txt                      G                            .  T                ATTAA                               1999
121-SD.txt                       A                            G  C                .....                               1999
Consensus      ttgcatgtttgtatgtac tccactctataatcaaacaattatcggg ac actttttatagaatta     caaggcgtaatagaccttgag
```

Supplementary Figure 8. Alignment of the upstream sequences of the GST_121 gene from susceptible and resistant strains of cotton bollworm.

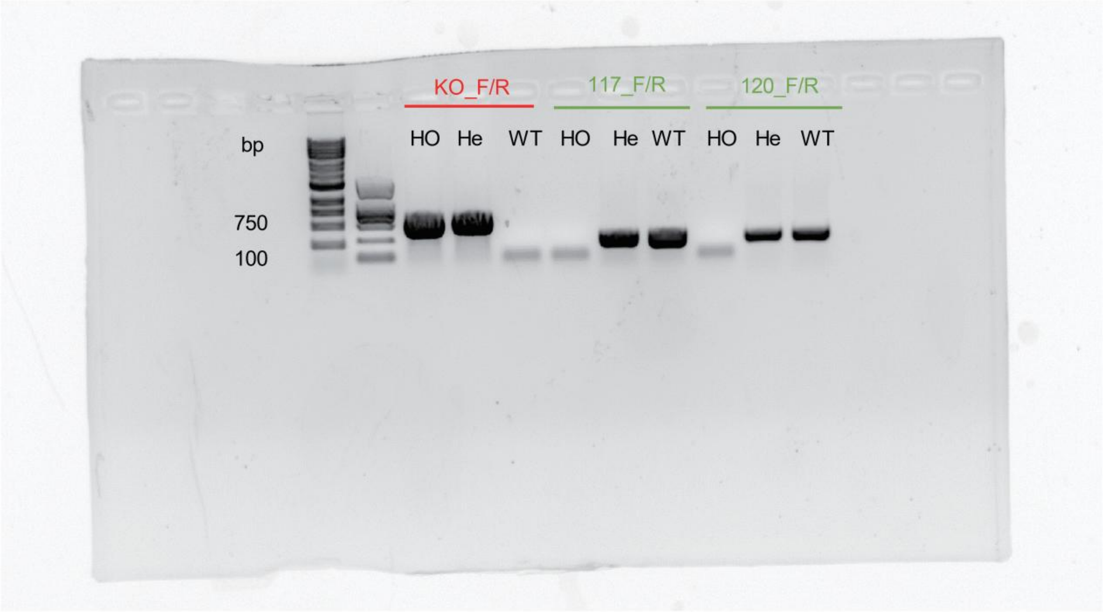

Supplementary Figure 9. Uncropped gel image for Figure 5b.

Supplementary Table 1. The primers used in this study.

Primer name	Sequence (5' to 3')	Purpose
GST_1F	GTTGAGTACGTGGATGTCAA	Genetic linkage
GST_1R	ATGCCGCTTTTAAAAGATGAT	
116-sgRNA	TGATCAGCACTTTCAACG	sgRNAs
121-sgRNA	ATTGGCCGACGAGCATCT	
KO_F	CAGCAGAACTTGGATAAA	Knockout detecting
KO_R	ATCCGTCAGGAGTGGTATCG	
117_F	ACATACCGTACCGACGTT	
117_R	GTGAGACACAATTCAGA	
120_F	CACGCCATTGCTGTATAT	
120_R	TAGTGGGATTCTGTTTAG	
q_PCR_116F	GTCATGCGATCATTACA	qPCR
q_PCR_116R	CAACACTAAGAGAGAC	
q_PCR_117F	CCTAATGTTGAGTATAT	
q_PCR_117R	TACCGACGTTGGTCGA	
q_PCR_118F	GAATCCTCAACACACTC	
q_PCR_118R	CAAATATGGAGCCGAGC	
q_PCR_119F	TCAGATGTTGAGTACG	
q_PCR_119R	TGCCGCTTTTAAAAGAT	
q_PCR_120F	GGTTTGACAGTATACAG	
q_PCR_120R	ACTATGTGAATGTCAACTTG	
q_PCR_121F	TATCATGGTCCTAACATTATA	
q_PCR_121R	AAATATGTTGAAACTGACC	
RPS3a-FP	GCGAAAATTCGTCGGAGACG	
RPS3a-RP	ATAATGATGATCTCCGAGCGA	
GAPDH-FP	AGATCGCTGTCTTCTGCGAG	
GAPDH-RP	CAGACGCCTTCTCTGTGGTT	
ds119_F	GCTAGTCCCCCTGTGAGGTCC	RNAi
ds119_R	TGATCAACGTCTCCACTTTGATA	
ds121_F	ATCATGGTCCTAACATTATAC	
ds121_R	CGTATTTGATGAATAAATAT	
Pro_NTC_119_F	TGCAAAGAGAGGAAGTAATAA	Promoter cloning
Pro_NTC_119_R	TATGTTTAAACCTTCTTTCAGAA	
Pro_XJ_119_F	GTTCTATTCCAAAAAAAACA	
Pro_XJ_119_R	AACCTTCTTTCAGACCGAAC	
Pro_NTC_121_F	AAAATCGCTGTTTCATTTACG	
Pro_NTC_121_R	GGCGTAATAGACCTTGAGG	
Pro_XJ_121_F	CAAATGCGATAGGAACAAAG	
Pro_XJ_121_R	AGGCGTAATAGACCTTGAGG	

Supplementary Table 2. Promoter sequences of GST_119 and GST_121 in XJ and NTC populations.

Name	Sequences
Promoter sequences of XJ_119	TAAGTTCTATTCCAAAAAAAACAACGTGAACGAAATAAAATTCTGGTTAGGT<br>TCCAAAAAAAATATTACAAATTAACTTGTCTTGCATGGTTCTAAAATACAATG<br>GTAACTTAATATGCAGAAAATATCGGATTCTCAATAAAGTCAGTATCAGAATT<br>TTTCTTCGTCCATATTCCTCTATCATAGTAGTCCAATGAGGTACATATTTTTTA<br>TTTATAGAAACGATGTCTTAACTAAAATAAAAGTTTTCTGGAAGTACGACGAT<br>AATTAATATAGGTTGTAAAAATATATCGTTTGTTAGAAGTGCCATTTTTTTGTC<br>TTACAGATACCCCAACCTCGTGGGCTGGTTGGAACGATGCGGGGAGCAAGA<br>GTTCTTTAAGAAGGGCAATGAACCCGGCTTGGAACAATTCCGGGATCTCAA<br>GAAATTGAAATTGGCCTAAGGCTAAAGATCTGCTCAGCAGTGTCTTCATGCC<br>TTTAAATACTGCTTTTACTAATATTTTGCGTTGCATTTCTTAAAGTAATATCTA<br>TATAAATTAATTGATTAAAAATTGTCTATGAAATCTTTATCATTAACATTAAATT<br>ATAACATAAATGCAGTTGTTTCATTTATTAAAAATCAGCGGAACAATAATATT<br>GTAGCTACAAACTACTTCTGCCCATAATAAAACGCCCATCCTTTTCATGTTTT<br>TGCTGGGAGGAAGAAGTGGTGGAACGAGAAAATAGCGTAATACACCAATAT<br>AACTACAAATCATAAAACAATACACCAATATAACTAGTAAACTACTTCTTATAC<br>CTACTAATAGTAATGTCCGAACTCATGTTTTTTAAGGAAGCACTAAACTGACA<br>GTACTCTTCGATTGTGGAGATTATTCAAATATCATCTATTAATATTGACAAGG<br>AAAAAGTTATACAAAATTGTTGCGATGACAAGCAGTAATAAGATCCGCATCAT<br>AAGTACAAAGATTTCGGTAGTGAAAACAATCATGCGATGCGAATTATCCACT<br>AACACCTGATATCTCCATCTTCTTCCTCCTGCCCTGCCCTGTTAACCATTTTT<br>TTTTAGATCGGCGTAATGTCTTTTGCTTCTACTCTTCTCTGTCACTCGTCGTA<br>CTGCCCCACTCACTCACTTCTTATTCATGTCACCTTTCAGGCAGTCCATGTC<br>CATCCATTTTTTCTAAGGTGTTCTTCTTCCTCTCCATCCATCTACTATCAGTT<br>GTTTATTAGATTGATGATTAGTTTATTGGTTTAGAGAAAATATAAAAAAATATA<br>ATGCCGTATCCATAACACACGGTTGGCTCGATAACAGTAAAAATACGTCCCA<br>CACGGACCGGCTTCAGCGGCCTGCGGCACTTCGGTCCACAACTTGAAGCGA<br>TTCGATCGTCTCTGCGAATAAGCGAGCTCGGATTTTCATTGGAACAAAATCA<br>AAAAGGTTATGTCAAATACAACAATGTGTTCAGAATGCTGGTGGTGCTGCCA<br>CGTTTTTGCAGAGCTTTGGGAATGTTCTCTGATCATCCGGAAGACGCTGTG<br>CCTCGTTTCTTCGCAGATTGCGTGACAGCTCCCACAGTATGCTGAGCACATT<br>GTGCGGTAGTTGGGATTGCCCATGGCTACGGCACTGGATCGGGCAGACACT<br>TCTGCGGATGTGTCATTATGGATTCATGGTCTGACAAGAATAAAATATTATTA<br>AACCTTCTTTCAGACCGAACCGACC
Promoter sequences of NTC_119	TGTGCAAAGAGAGGAAGTAATAATAATCACCAGCCTATCCTATTATCTACATC<br>TATGCGTACCCAGCCTATTACTAATGCGAAGAAAACTTAAAAATGGAGTAAC<br>TTCTCCAGCTTTCCCAACATTTCCCATCACTGTTCTGCTCCTATTGATCGTAG<br>CGTGATAAAAAGTATCCTATACCCTGCCCAGGAGTATAAAAAATATTAGTGCC<br>AAGTTTCATTAAAATCCGTCGAGTAGTTTTTGTTTCTATAACGAACATACAGA<br>CAGAAAGACAGACAGACAGAGAGCCAGACAGACAGACAGAAAGACAGACA<br>GACAGACAAAAATTTTACTCATTGCATTTTTGGCATCAGAATCGATCACTAAT<br>CACCCCCTGATAGATATTTTGAAAATATATTTAATGTACAGAATTGACCTCTA

	**CAGATTTATTATAAGTATAGATTCAAGAAGTAAAATAAAAAGCGGAACAATAT<br>TGTAGCTACAAACTACTTTTGCTCACATATAAAATGCCCATCATCATGGGAAG<br>AGAGTGGGGGAACGAACTTCCCATCAAAATGACTAGTCTCCACCAAGCCTT<br>TTCCCTCAAATATGGATGCCCATGGGTTTTTTTACCTGACATATTTTTGGGC<br>CATTTTAAAACGCCTATATGAGAAGAGAGAGAAGCAATTCTCCTTAAGCCAA<br>GTAGCCATTCATCTATAAAGCTAAGGGCATGTTTGGAAAATTATGTCAGTATT<br>AAATAGCCCAGCTTTAGAATCTGATAAAGCACAAGGTGGTGGTGGGCAAGG<br>TGGTGCTAGAGATATTTTGTCTGGAGATTTGTACTCACTAATAAGGTTATTTA<br>TTGTTGGAGTGCTTTGATGCAGAATTTCCTAGAATAGTTTCAGGCTTCGAAG<br>GGATATTTAGGCAAAAGGTCAAAAGGAAAAGCATCGGCCATTAGAGACGAA<br>GAAAAAAATACGGGGTAAGTCTTCACGAGAAGATTTTCAGTGTCTGTGTCA<br>GTTTGTCTGAAAGATTTCAACAGCTTTGCGGAGAAACTAGCATAATACATCA<br>ATAAACTACATAGCGTAAAACAAGACAACAAAATAACTAGTAAACTACTTCTT<br>ATATACTTACTAATAGTAATGTCCGAACTCACGTTTTTTAAGGAAGCACTAAA<br>TTGACAGTACTTATAGATTGTGGAGATTATTAAAATACCATCGATTAATATAGA<br>CAAGGAAAAAGTTATACAAAATGTACAAAGATTACAGTAATGAAAACAATCA<br>TGTGACACGAATTATCCACTAACAGCTGATATGCCCTGTTCAAAATTTTTTGG<br>GTCGGTGCAATGTCTTCCGCTTCCATTCTTGTCTGTCATTCGTCATACTGAC<br>TCTCACTTCTTTCTTATTCATGTCATCGTTCAGGCAGTCCATATCCATCCATT<br>TTTTCTTAGGTGTTTTTCTTCCTCTCCATCCATGATGATTAGCTTATTGGTTT<br>AGAGAAAATATAAAAAAATATAATGCCGTATCCATAACACACGGTTGGCTCGA<br>TAACGGTCACAATACGTCCCCACACGGACCGGCTTCAGCGGCCTACGGCAC<br>TTCAGTCTACAACTTGAAGCGATTCGATCGTAAGTGAGCTTGGATTTTCATT<br>TGAACAAAATCAAAAAGGTAATGTCCAATACAACAATGTGTTCAGAATGCTG<br>GTATACCACGTTTTTGCAGAGCTTCGGGAATATTCTCTGAGGTGAAGAATGA<br>TGGATTTATACCATCATCCGGAAGCGTTGTGCCTTGTTTCTTCGCAGATTGC<br>GTGACAGCTCCCACAGCATGCTGAGCACATTGTGCGGTGGGCTTGTCCATG<br>GCTGCGGCACTGGATCGGGCAGCACACATCTGCGGATGTATCCTACGTACT<br>CCGTAGATAGTGTGACATAGAGATTAGGCGTAAACGTAGACTTAATTTGATTA<br>AGTCTATTATAGATACATGGGTCTGACATAAATAAAATATGTTTAAACCTTCTT<br>TCAGAATAACA**
**Promoter sequences of XJ_121**	**CAAATGCGATAGGAACAAAGTAATTAAACTAGGCTTATGCTATTGACACTTTC<br>GTGTAAATACCACTGAGTATCATTTGTGGCTTGATTTTTGAGAGAATTCCAA<br>AAAGATTACACTTATTATGCTCAATATAGTCTAAACATAACCTGTAAACGACG<br>AAGATAGTATAAAAAATAATGTTTCACATGGATTGACAGTCTATGTGATTTTC<br>TAAGACTATACTCAAACTGCCAAACATTTACATAATTTAAATTGGTTCAGTAG<br>TTTTGGCGTGAAAAAGAGGCAAACGAACCAACAGAGATGTTTTACGCATACT<br>AGATGAAAAAATACGAACTGACAAACAAAAATTACCATTTCTAATGAAAAGC<br>GTTTATCCTGTTGGTAAAATCAATTATTCTAGTTGATCCAGATTTCGTGTAGA<br>AGTGTATACGTCAATCAATTTCTCATTATCGCGGCTGCCTATTCGAGCCAGG<br>CTTCTTGCAATATGCCATCATAATGCAATTTCCATGGAACTTTTAACGAGCAA<br>TTTCCTTGTCGATGTCTGCTTGCTTGGATTAATTAAATTTCCGTCTATACGTA<br>GTATGGGTCATATTATTAAAATATTTTGGCGTGGTTTCAGTGTGAGCGGGGC<br>TAAATATTTAAAATTGTTTTACTGGGTACTTAGCTACAGAAAACCTGCATCGT**

	**ACCACCAGCTATTGTACCACTCATACGAAAAGGTTCATCTTATATTATGGCTT<br>ATTATTTTCCGGGCGACTAATAACTTAAAAGTCGACAAATGTTTGCAACAAAT<br>AAAATAACTTGCCAGCGATTAAATATTTCGGCGACTATTATAATATGTACGCT<br>TTTTTTTTAATATTTTTTTTTTATTCACACTATGATTAATTTATATTCATTGAAA<br>TGTTTAACACTATTTTCTTAATTTAAATTCATTAAAATTTATTTTCTATTTTTTA<br>GTTCGGTTTTCTATAAAAAGCGTGTTTTTTAGTTTTTTTTTTAAACTATTATTA<br>ATTTTCTACTTTTTAGTTTGGTTTTTTATTAAAGCGTAATTTTTTAAAACTATT<br>ATTTGTTTTGTAGAATTAAAATTCTCTTTAGTCAATATTAGAGAAAACGGCTA<br>AAGATAATTTATTGGAAATAAAAATTAAAAACGAGTCCTGCCTTATCGACTGT<br>AGAGAAAAATTATAGAAAATTTTAATTAATTTTCTTACCTTATCGACTATATAT<br>GAAAATTATATAAATTAACAAAAATCATATTTTGCAAATTGAAAAACTTTTTTA<br>TTTTTCATTTCGTGCGTTGTGTAATCCCTATATCCCTTTCGATACAAAAGAAT<br>TTCTAAATAATTTGAAAGTGTATAAAAAGCTTTAAAACGTAAACAAGCACATA<br>CCCGCCTAAGTTTGTTTTATGGACCTAAAAATGACAATATTTTGGAAGCCATT<br>ATTTTGTTGCCATCTATTCACGCAAAGCTAAACTATATTTTTAGCGACCTCTA<br>TCGGCGAGTAGCAGAATCATGTTGATGATATAACTGGAGTTCACTGTGCTAG<br>TTTTGTTACTCGCCGCTAGATGTCTGACGTAAACGTTTTAATCGTAATTCAAG<br>ACTTCTCTAAACATTTTGTGGAGTGCGGTGTTCGTGTCTATTCTAGTAAGGA<br>TAACGCTTGGTTCGATCGCATTTTAATAGATGGCAGGCGATACGAAATACAC<br>AAATTTTGTCCAATGCATACATACAAAATGCACACTAACAGATAAATCTGTAG<br>TTAAAAAATTGAAAAAAAAAAACAACATCTTTCTTCTTATCGGTTCAACTTAA<br>CATCGAACTGAGAACTTCCTTTTTTTTAGTCGGCAAAAAATAACCCTTCTTG<br>ACCGTCCACTAATAAACAACATTCAGTATTTACTTACATTTTTTATTATATCTT<br>TGCATGTTTGTATGTACGTCCACTCTATAATCAAACAATTATCGGGACTACTT<br>TTTATAGAATTAATTAACAAGGCGTAATAGACCTTGAGG**
**Promoter sequences of NTC_121**	**CAAAATCGCTGTTTCATTTACGGCCAGTTTACTCACTTTTTCGGTGTTCTTAC<br>CGCAACACATTGAACACAAATACAGTCCACCTCCCACCGAGCACATAATCAT<br>AGTATAAAAATTAATGTTTCACATGGATGCCTCTGTAATTTCCTAAGACTTTA<br>CTCAAACTGACAAACATCTACATATAACAGCTACAATTGAATTTAAATTGGTT<br>CAGTAGTTATGGAAAAAAAAGCAGTGAAAAAGAGGCAGACGAACCAACAGA<br>GATATTTTATGCTCTTATAATACCTATTAGATGAAAAAATACGAACTGACAAA<br>CAAAAATTACCATCTCTAATGAAAAGCGTTTATCCTGCTGGTAAAATCAATTA<br>TTCTAGTTGATCCAGATTTCGTGTAGAAGTGTATACGTCAATCAATTTCTCAT<br>TATCGCGGCTGCCTATTCGAGCCAGGCTTCTTGCAATTTGCCATCATAATGC<br>AATTTCCATGGAACTTTTAACGAGCAATTTCCTTGTCGATGTCTGCTTGCTT<br>GGATTAATTAAATTTCCGTCTATACGTAGTATGGGTCATATTATTAAAATATTT<br>TGGCGTGGTTTCAGTGTGAGCGGGGCTAAATATTTAAAATTGTTTTACTGGG<br>TACTTAGCTACAGAAAACCTGCATCGTACCACCAGCTATTGTACCACTCATAC<br>GAAAAGGTTCATCTTATATTATGGCTTATTATTTTCCGAGCGACTAATAACTA<br>GTCGACAAATGTTTGCAACAAATAAAATAACTTGCCAGCGATTAAATATTTCG<br>GCGACTATTATAATATGTACGCTTTTTTTTAAAATATTTTTTTTTTATTCACAC<br>TGATTAATTTATATTCATTGAAATTTTTAACACTATTTTCTTAATTTAAATTCAT<br>TAAAATTTATTTTCTATTCGGTTTTCTATAAAAAGCGTGTTTTTTAGTTTTTTT<br>TTAAACTATTATTAATTTTCTACTTTTTAGTTTGGTTTTTTATTAAAGCGTAAT**

	**TTTTTAAAACTATTATTTGTTTTGTAGAATTAAAATTCTGTTTAGTCGATATTA<br>GAGAAAATGGCTAAAGATAATTTATTGGAAATAAAAATTAAAAACGAGTCCT<br>GCCTTATCGACTGTAGAGAAAAATTATAGAAAATTTTAATTAATTTTCTTACC<br>TTATCGACTATATATGAAAATTATATAAATTAACAAAAATCATATTTTGCAAATT<br>GAAAAACTTTTTTATTTTTCATTTCGTGCGTTGTGTAATCCCTATATCCCTTT<br>CGATACAAAAGAATTTCTAAATAATTTGAAAGTGTATAAAAAGCTTTAAAACG<br>TAAACAAGCACATACCCGCCTAAGTTTGTTTTATGGACCTAAAAATGACAATA<br>TTTTGGAAGCCATTATTTTGTTGCCATCTATTCACGCAAAGCTAAACTATATT<br>TTTAGCGACCTCTATCAGCGAGTAGCAGAATTATGTTGGTGATATAACTGGA<br>GTTCACTGTGCTAGGTTTGTTACTCGCCGCTAGATGTCTCACGTAAACGTTT<br>TAATCGTAATTCAAGACTTCTAAATAGAAACATTTTGTGGAGTTCGATGTTCG<br>TGTCTATCCTAGTAAGGATAACTTTTGGTTCGATCGCATTTTAATAGATGGCA<br>GGCGATACGAAGTACACCAATTTTGTCCAATGCATACATACAAAATGCACAC<br>TAACAGATAAATCTGTAGTTAAAAAATTGAAAAAAAAAACAACATCTTTCTTC<br>TTATCGGTTCAACTTAACATGTATATGTGCCTGGATACATACATAGGTAGGTA<br>CTTATAATATCTCGAACTGAGAACTTCCTTTTTTTTAGTCGGCAAAAAATAAC<br>CCTTCTTGACCGTCCACTAATAAACAACATTCAGTATTTACATTTTTTCTTATA<br>TCTTTGCATGTTTGTATGTACATCCACTCTATAATCAAACAATTATCGGGGAC<br>CACTTTTTATAGAATTACAAGGCGTAATAGACCTTGAGG**
